# Revealing the SARS-CoV-2 Spike Protein and Specific Antibody Immune Complex Formation Mechanism for Precise Evaluation of Antibody Affinity

**DOI:** 10.3390/ijms241713220

**Published:** 2023-08-25

**Authors:** Ieva Plikusiene, Vincentas Maciulis, Vilius Vertelis, Silvija Juciute, Saulius Balevicius, Arunas Ramanavicius, Julian Talbot, Almira Ramanaviciene

**Affiliations:** 1 NanoTechnas-Center of Nanotechnology and Materials Science, Faculty of Chemistry and Geosciences, Vilnius University, Naugarduko Str. 24, LT-03225 Vilnius, Lithuaniasilvija.juciute@chgf.stud.vu.lt (S.J.);; 2 State Research Institute Center for Physical Sciences and Technology, Saulėtekio Ave. 3, LT-10257 Vilnius, Lithuania; 3 Laboratoire de Physique Théorique de la Matière Condensée, Sorbonne Université, UMR 7600, 4 Place Jussieu, CEDEX 05, 75252 Paris, France

**Keywords:** SARS-CoV-2, spike protein, affinity interaction, immune complex formation, QCM–D, antigen–antibody binding kinetics

## Abstract

The profound understanding and detailed evaluation of severe acute respiratory syndrome coronavirus 2 (SARS-CoV-2) spike (SCoV2-S) protein and specific antibody interaction mechanism is of high importance in the development of immunosensors for COVID-19. In the present work, we studied a model system of immobilized SCoV2-S protein and specific monoclonal antibodies by molecular dynamics of immune complex formation in real time. We simultaneously applied spectroscopic ellipsometry and quartz crystal microbalance with dissipation to reveal the features and steps of the immune complex formation. We showed direct experimental evidence based on acoustic and optical measurements that the immune complex between covalently immobilized SCoV2-S and specific monoclonal antibodies is formed in two stages. Based on these findings it was demonstrated that applying a two-step binding mathematical model for kinetics analysis leads to a more precise determination of interaction rate constants than that determined by the 1:1 Langmuir binding model. Our investigation showed that the equilibrium dissociation constants (*K_D_*) determined by a two-step binding model and the 1:1 Langmuir model could differ significantly. The reported findings can facilitate a deeper understanding of antigen–antibody immune complex formation steps and can open a new way for the evaluation of antibody affinity towards corresponding antigens.

## 1. Introduction

The many health challenges arising from Severe Acute Respiratory Syndrome Coronavirus 2 (SARS-CoV-2) resulted in intensive studies of SARS-CoV-2 spike protein (SCoV2-S) and specific antibodies [1,2,3]. The key task in this field is the recognition of viral surface proteins by high-affinity reagents that represents a promising strategy for virus detection or neutralization. For this reason, in many papers focusing on SARS-CoV-2 properties research, a great amount of attention was paid to the determination of the equilibrium dissociation constant (*K*_D_) of the interactions between the receptor and the spike protein as well as the spike protein and the neutralizing antibodies [4,5,6,7]. In the case of the SARS-CoV-2 structural protein and specific antibody interaction, the *K*_D_, which is determined as a ratio of dissociation (*k*_d_) and association (*k*_a_), rate constants can range from pM up to hundreds of nM. The *K*_D_ is calculated by analyzing protein binding or interaction kinetics measured experimentally in real time. Commonly, techniques for biomolecular interactions and determination of *K*_D_ are used: microscale thermophoresis, isothermal titration calorimetry, biolayer interferometry, and surface plasmon resonance (SPR) [8,9,10,11]. Some of them have limitations in measuring high-affinity-antibody interaction with immobilized antigens [12]. Spectroscopic ellipsometry (SE) enables us not only to measure the amplitude change of the light wave after the reflection from the surface similarly to SPR but, in addition, it can provide information on the phase shift that is much more sensitive to the refractive index changes at the solid–liquid interface. In the case of applying SE for antigen–antibody interaction study, it is possible to measure two parameters: (i) *Ψ*, which corresponds to light wave amplitude, and (ii) Δ, which represents phase shift evolution in time. As was previously demonstrated in our work, the ellipsometric parameter Δ(*t*) has much higher sensitivity in the initial phase of antigen–antibody immune complex formation, while the parameter *Ψ*(*t*) more sensitive when the steady-state conditions are reached [13]. The advantage of a label-free SE method is that it can be simultaneously combined with another highly surface-sensitive method QCM–D and this allows us to investigate not only optical but also mechanical properties of the layer within the same experiment and obtain a more detailed analysis of the immune complex formation in real time. Usually, proteins including SCoV2-S and specific antibody interactions are measured by SPR and analysis is performed by applying the 1:1 Langmuir mathematical binding model [14]. But in some cases, when the binding of specific antibodies to target proteins is strong and can be treated as firmly bound and intermediate complex formation takes place, this model for kinetics constants measurements can fail [15,16]. For this purpose, the investigation of SCoV2-S and specific antibody immune complex formation in real time, using label-free, non-contact kinetics measurement methods that can be applied simultaneously and independently attracts attention as each technique give different information during the same measurement and prove the immune complex formation steps. Protein adsorption or covalent immobilization on a surface is often accompanied by conformational changes that can be reversible or irreversible, leading to the formation of rigid or viscoelastic layers [17,18]. The viscoelastic properties of protein layers found in the lungs [19] and saliva [20] are important in the physiological functions of the organism. Knowledge of the properties of mAbs, particularly the viscosity and elasticity, is important for the evaluation and a deeper understanding of the process of immune complex formation and application in drug design. The mAbs agains SARS-CoV-2 can block the virus from entering and infecting cells. All mAbs bind to the same epitopes of antigens in contrast to polyclonal antibodies [21]. The mAbs are used to treat numerous health problems including arthritis, Crohn’s disease, chronic plaque psoriasis, cryopyrin-associated periodic syndromes, cancers, and postmenopausal osteoporosis [22,23,24,25].

Promising tools for this type of study are non-contact, real-time and surface-sensitive optical and acoustic methods, which enable one to study the proteins’ behavior at the solid-liquid interface. The former category includes highly sensitive SE, which can determine the evolution of protein monolayer mass via measurement of refractive index and evaluate the thickness of the monolayer [16,26]. SE, in its total internal reflection mode, was successfully applied to evaluate SARS-CoV-2 nucleoprotein immobilization and interaction with specific polyclonal antibody kinetics and the associated thermodynamic parameters [15]. The other contactless, precise, time-resolved and ultra-mass sensitive method is quartz crystal microbalance with dissipation (QCM–D). It uses acoustic waves by measuring shifts in frequency (Δ*F*) and energy dissipation (Δ*D*) during the formation of the protein monolayer. This method can also evaluate the surface mass and give information about the viscoelastic properties of the protein monolayer [27,28]. The application of combined SE and QCM–D techniques during one measurement enabled the study of SARS-CoV-2 structural proteins as a nucleocapsid monolayer formation and interaction with a specific antibody in real time and provided information about polyclonal antibody flexibility [29]. The advantage of this combined technique is that it can provide a more precise determination of the surface mass density and viscoelastic properties. Moreover, further combining these results from optical-acoustic study allows us to obtain experimental evidence of the immune complex formation steps and support mathematical modeling results.

In this work, we present a real-time analysis of the SARS-CoV-2 SCoV2-S protein covalent immobilization at a solid-liquid interface and affinity interaction with specific mAb-SCoV2-S using a combination of SE and QCM–D. The experimentally obtained results were further analyzed using a two-step binding mathematical model, which can be applied when an intermediate immune complex is formed during antibody and antigen interactions. The goal of the present study was to demonstrate how information about dielectric properties of the proteins (SCoV2-S and mAb-SCoV2-S) used as monolayers in the model system can be combined with viscoelastic properties and how calculated viscosity and elasticity can validate two-step binding mathematical model eligibility for precise calculation of affinity constants. The current application of the methods and the proposed model is not limited to SCoV2-S protein and mAb-SCoV2-S antibody monolayer model systems and can be further applied for any kind of protein monolayer characterization.

## 2. Results and Discussion

The covalent immobilization of SCoV2-S on 11-MUA self-assembled monolayer (SAM) and the subsequent affinity interaction between SCoV2-S and mAb-SCoV2-S were monitored simultaneously using SE and QCM–D techniques.

After reaching steady-state SE and QCM–D signals during both processes, (i) covalent immobilization of SCoV2-S and (ii) the interaction of immobilized SCoV2-S with mAb-SCoV2-S, a washing procedure based on the surface treatment with 0.01 M PBS solution, pH 7.4, was applied. It was determined that in both cases the washing procedure did not induce any changes in any registered SE and QCM–D signals demonstrating that SCoV2-S was well immobilized to 11-MUA carboxyl groups and mAb-SCoV2-S were stronglybound to immobilized SCoV2-S.

### 2.1. The Determination of Viscoelastic Properties

**Figure 1:**
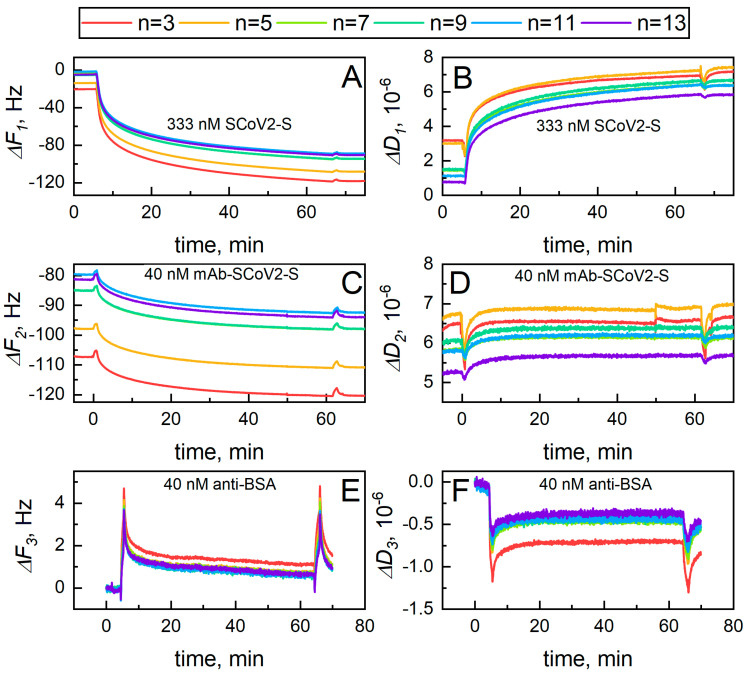
Real-time monitoring of Δ*F*_1_ and Δ*D*_1_ evolution in time for covalent immobilization of SCoV2-S on 11-MUA SAM (**A**,**B**) and Δ*F*_2_ and Δ*D*_2_ for affinity interaction between SCoV2-S and mAb-SCoV2-S (**C**,**D**), and SCoV2-S interaction with anti-BSA antibodies (**E**,**F**). Each curve corresponds to a particular harmonic, n.

The protein monolayer viscosity and elasticity were determined in the following way. The first step was the determination of the frequency Δ*F*_s_ and dissipation Δ*D*_s_ change when a QCM–D sensor disc covered by 11-MUA SAM was placed into PBS solution. The second step was the measurement of Δ*F*_sc_ and Δ*D*_sc_ kinetics during the covalent immobilization of SCoV2-S and the formation of a monolayer on the surface. During the last step, Δ*F*_scm_ and Δ*D*_scm_ changes corresponding to a SCoV2-S/mAb-SCoV2-S immune complex formation was measured. Figure 1A,B shows the kinetics of (Δ*F*_sc_ − Δ*F*_s_) = Δ*F*_1_ and (Δ*D*_sc_ − Δ*D*_s_) = Δ*D*_1_, which represent the covalent SCoV2-S immobilization and monolayer formation process, respectively. Figure 1C,D represents the (Δ*F*_scm_ − Δ*F*_sc_) = Δ*F*_2_ and (Δ*D*_scm_ − Δ*D*_sc_) = Δ*D*_2_ kinetics obtained when mAb-SCoV2-S monolayer was formed onto SCoV2-S.

During the initial phase of SCoV2-S covalent immobilization (Figure 1A,B), the Δ*F*_1_ and Δ*D*_1_ signals shifted rapidly, reaching 50% of the total signal value (50 Hz for 3rd harmonic) in the first 3 min; then, as SCoV2-S immobilization continued, the system saturated, slowing the evolution of the Δ*F*_1_ and Δ*D*_1_ signals. After covalent immobilization of SCoV2-S, the Δ*F*_1_ values changed in the range of 90–100 Hz, depending on the monitored harmonic, with the lower harmonics (3rd, 5th) exhibiting higher change in Δ*F*_1_. The Δ*D*_1_ values are positive when monitoring a layer formation, and range from 4 × 10^−6^ to 5 × 10^−6^, depending on the harmonic measured, with the lower harmonics exhibiting a lower change in Δ*D*_1_. These values indicate that a layer consisting of covalently immobilized SCoV2-S has a significant amount of hydrodynamically trapped PBS solution [30]. Figure 1C,D shows the QCM–D signal change for the formation of the SCoV2-S/mAb-SCoV2-S immune complex. After the formation of the SCoV2-S/mAb-SCoV2-S immune complex, the Δ*F*_2_ values for all monitored harmonics were ~13 Hz, while the Δ*D*_2_ values increased in the range of 0.2 × 10^−6^–0.4 × 10^−6^. Injection of PBS solution after immune complex formation did not cause dissociation of the proteins (no significant increase in frequency was observed), i.e., the monoclonal antibodies that entered the chamber formed a stable complex with the immobilized SCoV2-S. The control experiment for the evaluation of nonspecific binding of antibodies was performed by injecting 40 nM of anti-BSA antibodies for interaction with immobilized SCoV2-S (Figure 1E,F). All other experimental conditions were the same as using mAb-SCoV2-S.

**Figure 2:**
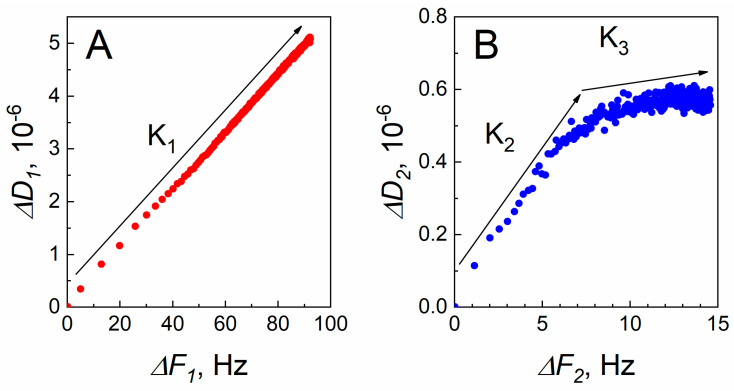
Δ*D* vs. Δ*F* plots for covalent immobilization of SCoV2-S (**A**) and SCoV2-S/mAb-SCoV2-S immune complex formation (**B**). The arrows K_1_ = 0.0548; K_2_ = 0.0624; K_3_ = 0.0065 indicate the slopes to red and blue curves.

The Δ*D* vs. Δ*F* plots reveal different time dependencies [30]. By removing time as a parameter, the slope of the curve shows how much dissipation is caused by an additional unit of Δ*F* (mass). However, time can be inferred by the density of data points in the Δ*D* vs. Δ*F* plots. Figure 2A,B represents the plots for SCoV2-S covalent immobilization and Δ*D*_2_ vs. Δ*F*_2_ for SCoV2-S/mAb-SCoV2-S immune complex formation, respectively.

A Δ*D*_1_ vs. Δ*F*_1_ plot of the covalent immobilization of SCoV2-S, presented in Figure 2A, shows a nearly linear behavior [30,31] with a calculated slope K_1_ = 0.0548. As can be seen from Figure 2A, the points are further apart in the region from 0 to 40 Hz. This corresponds to a fast immobilization rate on an activated surface with a large number of immobilization sites. In the interval from 40 to 90 Hz, the rate of immobilization slows down as the coverage of SCoV2-S increases, and it is seen by densely placed data points. The linear behavior of the Δ*D*_1_ vs. Δ*F*_1_ plot in Figure 2A is consistent with the formation of a SCoV2-S monolayer with no significant conformational changes, which is typically observed during the immobilization of globular proteins [31,32]. The formed monolayer of SCoV2-S can be described as dissipative and laterally heterogeneous [33]. The total dissipation value is 5 × 10^−6^ and can be attributed to the movement of protein molecules, with dissipation mainly occurring at the boundary between SCoV2-S and PBS solution [34].

The Δ*D*_2_ vs. Δ*F*_2_ plot in Figure 2B for the formation of the SCoV2-S/mAb-SCoV2-S immune complex is more intricate than for the covalent immobilization of SCoV2-S and exhibits two linear regimes. The initial one, with slope K_2_ = 0.0624, shows a higher Δ*D*_2_ per unit of Δ*F*_2_ and a faster SCoV2-S/mAb-SCoV2-S interaction rate (is reflected by points further apart). Compared to the following regime, with slope K_3_ = 0.0065, where the Δ*F*_2_ change during the interaction between SCoV2-S and mAb-SCoV2-S does not significantly increase the Δ*D*_2_ values, the interaction rate is slower (points are closer to each other). The initial increase in Δ*D*_2_ can be associated with molecular rearrangement of surface-bound SCoV2-S upon injection of mAb-SCoV2-S [35], as well as with conformational changes of surface-bound mAb-SCoV2-S antibody (Fab fragments flexibility, changes in Fab-Fab angles, movements of side-chains or some structural changes of the antibody CDR loops). The differences in Δ*D* vs. Δ*F* traces that are related to the processes taking part at the sensing surface were also reported by other authors investigating the reduced antibody fragments interaction with bovine leukemia virus antigen gp51 [35], bovine serum albumin and specific antibodies [31], and other proteins adsorption and cells adhesion [32]. The higher Δ*D*_2_ vs. Δ*F*_2_ ratio shows that at the initial phase of SCoV2-S and mAb-SCoV2-S interaction more flexible SCoV2-S/mAb-SCoV2-S immune complexes are formed. The same tendency at the initial phase of immune complex formation with the following slight change of slope was observed with SARS-CoV-2 nucleocapsid protein and specific polyclonal antibodies [29]. The change of Δ*D*_2_ vs. Δ*F*_2_ slope (Figure 2B) in the 7–8 Hz range could be related to decreasing amount of water after firm bivalent binding of mAb-SCoV2-S, producing a more rigid and unyielding layer of the SCoV2-S/mAb-SCoV2-S immune complexes [36]. In contrast, during non-specific interaction between bovine serum albumin and gp51, a gradual increase in dissipation was observed from the initial stage [35].

### 2.2. The Assessment of Optical Properties

**Figure 3:**
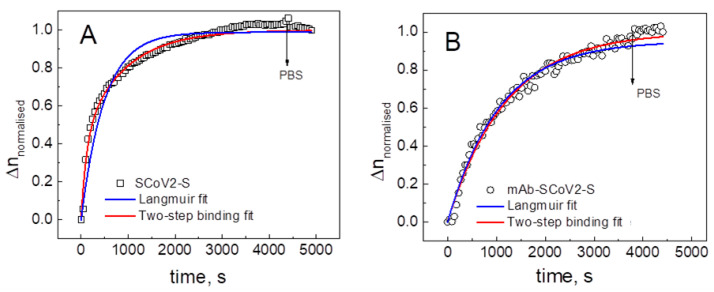
Time evolution of the normalized refractive index during immobilization of SCoV2-S on 11-MUA SAM (**A**) and SCoV2-S/mAb-SCoV2-S immune complex formation (**B**); dots and squares correspond to experimental points and curves to the fit of the mathematical model.

Using the SE/QCM–D technique during the covalent immobilization and immune complex formation acoustic and optical signals were registered simultaneously. It enables us to measure the normalized refractive index (Δ*n*_normalized_ = Δ*n*(t)/Δ*n*_max_) evolution in time (here Δ*n*_max_ is maximal refractive index change) for SCoV2-S immobilization kinetics (A) and SCoV2-S/mAb-SCoV2-S immune complex formation kinetics (B). The results of these investigations are presented in Figure 3A,B.

In order to obtain the surface mass density of covalently immobilized SCoV2-S monolayer and bound mAb-SCoV2-S monolayer it is necessary to calculate the refractive index and thickness of the layers using regression analysis. The applied optical model consisted of a QCM–D gold-coated substrate with thickness *d_Au_*= 200 nm coupled with a self-assembled monolayer of 11-MUA SAM with thickness *d_SAM_* = 0.9 nm [37]. Taking into account the dimensions of the SCoV2-S protein [6] and the disordered nature of the SCoV2-S protein immobilization on 11-MUA SAM, SCoV2-S monolayer thickness of *d_SCoV2-S_* = 12.93 nm was calculated from regression analysis. The gold-coated substrate of QCM–D was modeled with a B-Spline function with gold as a starting material from the Complete EASE software database (version 5.08gsc). The 11-MUA SAM was modeled using Cauchy dispersion [38] with corresponding parameters A = 1.491; B = 0.006. The SCoV2-S monolayer was modeled using the Bruggeman effective media approach (EMA) with two components: SCoV2-S and PBS solution. Both SCoV2-S and PBS solution components were modeled using Cauchy dispersion A = 1.572; B = 0.010 for SCoV2-S and A = 1.318; B = 0.005 for PBS. During the regression analysis, the thickness of the SCoV2-S monolayer was fixed, while the SCoV2-S and PBS solution percentage in EMA was the free-fitted value resulting in 20.7% of SCoV2-S and 79.3% of PBS solution for a fully formed monolayer. The formation of the SCoV2-S/mAb-SCoV2-S immune complex was modeled using an analogous Bruggeman EMA, with the fraction of PBS solution in the mAb-SCoV2-S monolayer as the fitting parameter. The calculations showed that the fully formed monolayer of mAb-SCoV2-S consisted of 3.8% mAb-SCoV2-S and 96.2% PBS. After that, the surface mass density (*Γ^SE^*) of both the SCoV2-S and mAb-SCoV2-S monolayer was calculated using the de Feijter formula [39]:

(1) $$\Gamma^{SE}=\frac{d(n_{layer}-n_{PBS})}{dn/dc}\cdot 100$$

Here, *d* is the thickness of the formed monolayer (SCoV2-S or mAb-SCoV2-S), *dn*/*dc* is the refractive index increment (for globular proteins it is estimated as 0.18 [40]), *n_layer_* and *n_PBS_* are the refractive indices of the SCoV2-S or mAb-SCoV2-S monolayer and the surrounding PBS solution, respectively.

### 2.3. The Evaluation of SCoV2-S and mAb-SCoV2-S Monolayers Hydration

Since the SCoV2-S monolayer exhibits high dissipation (Δ*D* ≥ 1 × 10^−6^), it is possible to use QCM–D data for analysis based on the Voigt–Voinova viscoelastic model and calculate the surface mass density (*Γ^QCM−D^*) of SCoV2-S.

The *Γ^QCM−D^* is usually referred to as wet mass, whereas *Γ^SE^*, which does not include PBS solution, is referred to as dry mass. So the fraction of PBS solution (*f*_PBS_) inside the SCoV2-S monolayer, also referred as hydration, can be calculated using the following formula [27]:

(2) $$f_{PBS}=1-\frac{\Gamma^{SE}}{\Gamma^{QCM-D}}$$

**Figure 4:**
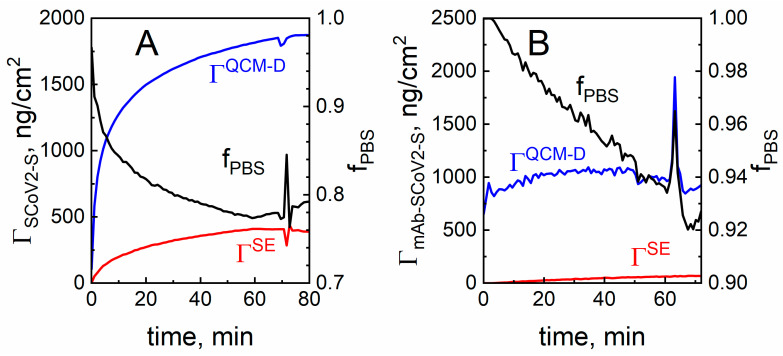
The surface mass density and PBS solution fraction (hydration) evolution in time for SCoV2-S covalent immobilization (**A**) and for the immune complex of SCoV2-S/mAb-SCoV2-S formation obtained using QCM–D and SE (**B**).

Here, *f*_PBS_ is the fraction of PBS solution in the formed protein (SCoV2-S or mAb-SCoV2-S) layer, *Γ^SE^*—dry mass, *Γ^QCM−D^*—wet mass of SCoV2-S or mAb-SCoV2-S. The *f*_PBS_ of both SCoV2-S and mAb-SCoV2-S monolayers calculated from the combined SE/QCM–D measurements and Equation (2) is shown in Figure 4A,B. As it can be seen from this figure, the maximal surface mass density after full covalent immobilization of SCoV2-S calculated from SE was *Γ^SE^* = 388 ng/cm^2^, while the density obtained from QCM–D analysis was *Γ*^QCM−D^ = 1873 ng/cm^2^. The difference is due to the QCM–D method sensitivity to PBS solution influence. The corresponding fractions of PBS were *f*_PBS_ = 0.792 for the covalently immobilized SCoV2-S monolayer and *f*_PBS_ = 0.962 for mAb-SCoV2-S, respectively.

### 2.4. The Determination of Shear Viscosity and Elasticity of Formed SCoV2-S and mAb-SCoV2-S Monolayers

For the calculation and analysis of shear viscosity coefficient (*η*) and shear elasticity modulus (*μ*) kinetics, we used the Voigt–Voinova model [41], which describes viscoelastic properties of two layers covering the surface of a piezoelectric sensor oscillating in a pure shear mode in the bulk liquid. We consider these layers to be the SCoV2-S and mAb-SCoV2-S monolayers, correspondingly. According to this model, the η and μ are related to changes in frequency Δ*F* and dissipation factor Δ*D*, which are experimentally measurable values. We adapted the analytical expressions of Δ*F* and Δ*D* presented in reference [41] to the experimental results used in our work by omitting terms representing the influence of the bulk liquid because the first step of the measurement was dedicated recording of QCM–D response before the formation of SCoV2-S/mAb-SCoV2-S immune complex. As a result, the following Equations for Δ*F* and Δ*D* are obtained:

(3) $${∆F}_{j}=-\frac{1}{2\pi \rho_{0}d_{0}}\sum_{j}\left[d_{j}\rho_{j}\omega -2h_{j}{\left(\frac{\eta_{3}}{\delta_{3}}\right)}^{2}\frac{\eta_{j}\omega^{2}}{\mu_{j}^{2}+\omega^{2}\eta_{j}^{2}}\right]$$

(4) $${∆D}_{j}=\frac{1}{2\pi f\rho_{0}d_{0}}\sum_{j}2d_{j}{\left(\frac{\eta_{3}}{\delta_{3}}\right)}^{2}\frac{\mu_{j}\omega }{\mu_{j}^{2}+\omega^{2}\eta_{j}^{2}}$$

Here, *ρ*_0_ and *d*_0_ are the density and thickness of the oscillating quartz crystal, respectively, *ω* = 2π*·f* where *f* is the resonant frequency, *δ*_3_ = (2 *η*_3_/*ρ*_3_ *ω*)^1/2^ where *η*_3_ and *ρ*_3_ are the viscosity and density of the PBS solution.

We omitted terms representing the influence of the bulk liquid because the first step of the measurement was to record the QCM–D response before the formation of SCoV2-S and mAb-SCoV2-S monolayers. The Δ*F* and Δ*D* values corresponding to this response were always excluded from a total QCM–D signal in order to investigate only the viscoelastic properties of SCoV2-S and mAb-SCoV2-S. The summation in Equations (3) and (4) is carried out over all viscoelastic layers between the quartz crystal and the bulk liquid. The index *j* = 1, and *j* = 2 for SCoV2-S and mAb-SCoV2-S monolayers, respectively. The corresponding *η*_j_ and *μ*_j_ values can be calculated using Equations (5)–(8) and (3)–(4).

(5) $$\eta_{j}=\frac{A_{j}}{B_{j}^{2}+A_{j}^{2}}$$

(6) $$\mu_{j}=\omega \sqrt{\frac{1}{A_{j}}\eta_{j}-\eta_{j}^{2}}$$

(7) $$A_{j}=\frac{\rho_{0}d_{0}{∆F}_{j}+{\rho_{j}d}_{j}f}{\eta_{3}\rho_{3}d_{j}f}$$

(8) $$B_{j}=\frac{\rho_{0}d_{0}{∆D}_{j}}{\rho_{3}d_{j}\eta_{3}}$$

**Figure 5:**
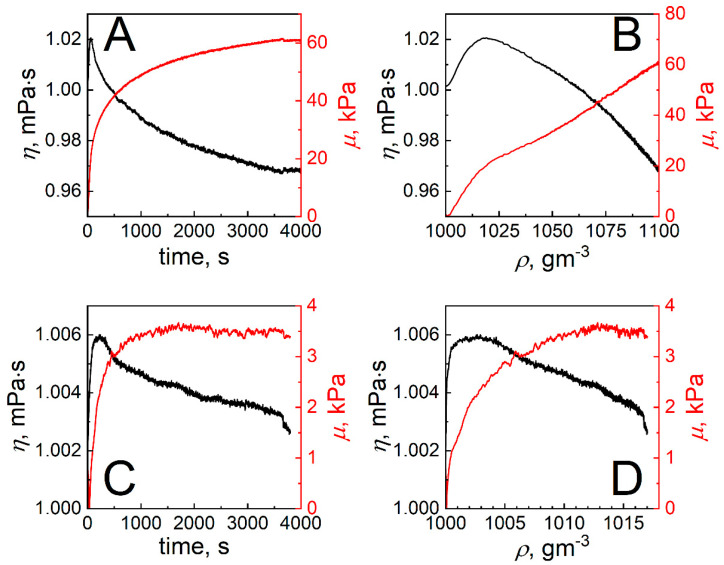
Viscosity coefficient (*η*) (black) and shear elasticity modulus (*μ*) (red) vs. time and effective layer density (*ρ* = *ρ_1_*) for SCoV2-S covalent immobilization (**A**,**B**) and for mAb-SCoV2-S affinity interaction (**C**,**D**) (*ρ* = *ρ_2_*).

In this case, it is reasonable to assume that during the formation of SCoV2-S or mAb-SCoV2-S monolayers, the main factor influencing the change of refractive index (*n*) and the mass of the immobilized molecules is the effective density of SCoV2-S or mAb-SCoV2-S monolayers *ρ*_j_, assuming that the thickness of the layers *h*_j_ is constant. The dependencies *n* vs. time (*t*) obtained using this approach are presented in Figure 3A,B. We then determined the *η* and *μ* of SCoV2-S and mAb-SCoV2-S monolayers as a function of time (*t*) as well as a function of the monolayer effective density *ρ* = *ρ*_j_. The results of these calculations are presented in Figure 5.

As can be seen from Figure 5A,C, *μ* increases with time during the formation of both SCoV2-S and mAb-SCoV2-S monolayers. The fully formed SCoV2-S monolayer is, however, more rigid than the mAb-SCoV2-S one. This is a result of significantly stronger covalent binding of SCoV2-S molecules to the 11-MUA SAM in comparison to the weaker, affinity interaction of SCoV2-S and mAb-SCoV2-S in the consequently binding state. Moreover, the dependencies of *μ* on *ρ* of both SCoV2-S and mAb-SCoV2-S monolayers are different (Figure 5B,D). In the case of SCoV2-S, one observes two well-defined linear regions with different slopes, while for the mAb-SCoV2-S monolayer *μ* is a nonlinear function of *ρ* over the entire range. The *η* of both monolayers increases with *t* and *ρ* at the beginning of the process and after reaching maximum value gradually decreases. We note that for the SCoV2-S monolayer, the value of *η* at the end of monolayer formation is less than the viscosity of PBS solution (*η*_3_). However, in the case of the mAb-SCoV2-S monolayer, *η* decreases with *t* and *ρ*, although it always remains higher than *η*_3_.

**Figure 6:**
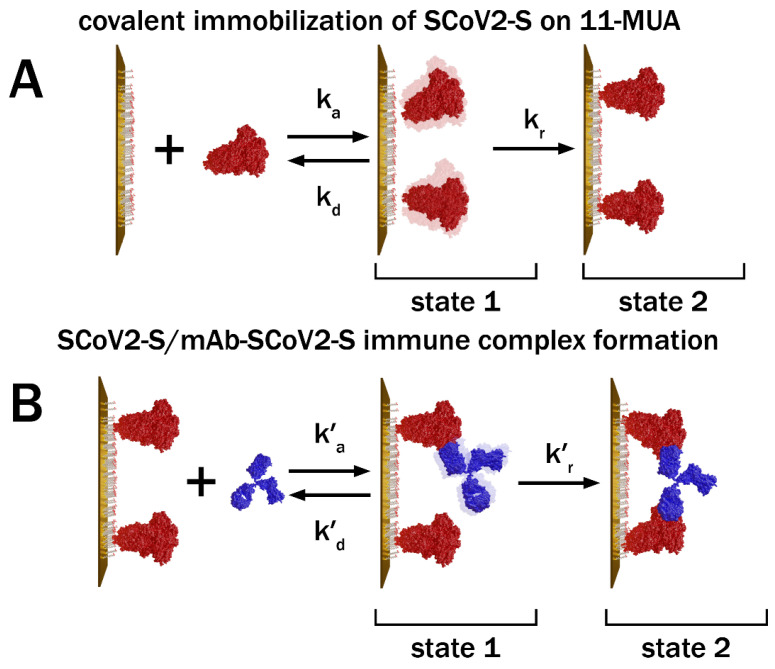
Schematic representations of: the two-step model of SCoV2-S covalent immobilization (**A**) and affinity-based interaction between covalently immobilized SCoV2-S and mAb-SCoV2-S (**B**). The proteins enter the initial state 1 at a rate constant *k*_a_, then may desorb at a rate constant—*k*_d_ or bind strongly at rate constant—*k*_r_.

The changes in the viscoelastic properties of the SCoV2-S and mAb-SCoV2-S monolayers during covalent immobilization and immune complex formation can be explained by assuming that the total number of proteins immobilized on the 11-MUA SAM or bounded after affinity interaction to the SCoV2-S monolayer at each time instant is a sum of temporarily associated and irreversibly bound molecules. At the beginning of the monolayer formation process, a large part of the monolayer consists of temporarily associated molecules, because the association rate of molecules is significantly higher than the rate of their consequently binding. An increase of SCoV2-S or mAb-SCoV2-S concentration on the surface during the initial formation period leads to increased viscosity and elasticity, as is typically observed in protein solutions [42]. The subsequent growth of the protein monolayer is accompanied by the formation of strongly bound sites, that have an impact on the total monolayer mass increase in time (Figure 4). Finally, the fully formed protein monolayer consists mainly of these strongly and irreversibly bound molecules (Figure 6, state 2). The number of strongly bonded molecules gradually increases during the whole monolayer formation process, while the number of weakly associated proteins increases at the beginning of monolayer formation and then significantly decreases (Figure 5). In comparison with the buffer solution, the irreversibly bound protein molecules have higher elasticity but lower viscosity (Figure 5). As the elasticity component during the formation of the monolayer increases all the time, the effective elasticity *μ* also increases in time. However, the viscosity *η* vs. time dependence exhibits a local maximum. Thus, it can be concluded that the protein monolayer formation process takes place via a two-step mechanism. In the first step before the final irreversible immobilization of SCoV2-S on the 11-MUA SAM or stable SCoV2-S/mAb-SCoV2-S immune complex formation molecules are weakly bonded to the target with the possibility of returning to the PBS solution due to the dissociation process, while in the second step they are bounded strongly.

### 2.5. The Modelling of Covalent SCoV2-S Immobilization and Affinity Interaction with mAb-SCoV2-S Antibodies Kinetics

For modeling of SCoV2-S immobilization and affinity interaction with mAb-SCoV2-S antibodies kinetic and calculation of association and dissociation rate constants, the two-step binding model was applied [13,15,43]. This model takes into account the formation of the encounter immune complex between the immobilized antigen and specific antibody. After the encounter complex is formed in state 1 (with rate constant *k_a_*), it may become fixed (rate constant *k_r_*), resulting in a stable antigen–antibody complex in state 2, or both of the molecules may drift apart (at rate constant *k_d_*) [44]. The schematic representation of the stages of covalent SCoV2-S immobilization and the immune complex formation are presented in Figure 6.

According to this model, in the initial stage of covalent immobilization of SCoV2-S or affinity interaction of mAb-SCoV2-S proteins are in state 1. This state is reached with the rate *k*_a_ × *c* (here *c* is the concentration of SCoV2-S or mAb-SCoV2-S in PBS solution and *k*_a_ is the association rate constant). Upon reaching state 2, SCoV2-S (during covalent immobilization) or mAb-SCoV2-S (during affinity interaction) can desorb from the surface at a rate constant—*k*_d_, or become strongly bounded at a rate constant—*k*_r_. At the beginning of the SCoV2-S covalent immobilization, the SCoV2-S monolayer consists mainly of molecules in state 1. Later, however, they transform to state 2 and finally the monolayer consists of only irreversibly bound SCoV2-S. The same scenario applies to mAb-SCoV2-S during affinity interaction with covalently immobilized SCoV2-S. The two-step consequent binging process is described by the following system of differential equations:

(9) $$\frac{{dr}_{1}}{dt}=k_{a}c\left(1-r_{1}-r_{2}\right)-\left(k_{d}+k_{r}\right)r_{1}$$

(10) $$\frac{dr_{2}}{dt}=k_{r}r_{1}$$

(11) $$r=r_{1}+r_{2}=1+\left(Me^{-f_{+}t}-Ne^{{-f}_{-}t}\right)$$

Here *r*_1_ and *r*_2_ are normalized concentrations of proteins in state 1 and state 2 (Figure 6), respectively. The solution of Equations (9) and (10) that lead to (11) can be used to calculate the total concentration (*r*) of bounded molecules.

Here *f*_+_, *f*_−_, *M* and *N* are:

(12) $$f_{\pm }=\frac{\left(k_{d}+k_{r}+k_{a}c\right)\pm \sqrt{{\left(k_{d}+k_{r}+k_{a}c\right)}^{2}-4k_{a}ck_{r}}}{2};\;M=A\left(1-\frac{k_{r}}{f_{+}}\right);\;N=A\left(1-\frac{k_{r}}{f_{-}}\right);\;A=\frac{k_{a}c}{\sqrt{{\left(k_{d}+k_{r}+k_{a}c\right)}^{2}-4k_{a}ck_{r}}}$$

Fits of this model to experimental data assuming that *r* = Δ*n*_normalized_ are presented in Figure 3. The best-fit parameters for the association, dissociation, and consequent binding kinetic coefficients are *k*_a_ × *c* = 3.8 × 10^−3^ s^−1^; *k*_d_ =1.2 × 10^−3^ s^−1^ *k*_r_ =2.6 × 10^−3^ s^−1^ for SCoV2-S covalent immobilization and *k′*_a_ × *c* = 1.6 × 10^−3^ s^−1^; *k′*_d_ = 2.5 × 10^−3^ s^−1^ *k′*_r_ = 3.0 × 10^−3^ s^−1^ for mAb-SCoV2-S affinity interaction with SCoV2-S. The *K*_D_’s are 107 nM and 62 nM for SCoV2-S and mAb-SCoV2-S, correspondingly.

The two-step binding model better fits the data than with the conventional 1:1 Langmuir binding model (see Figure 3). The kinetic coefficients were *k*_a_ × c = 2.0 × 10^−3^ s^−1^; *k*_d_ = 2.1 × 10^−5^ s^−1^ for SCoV2-S covalent immobilization and *k*_a_ × c = 9.0 × 10^−4^ s^−1^; *k*_d_ = 4.5 × 10^−5^ s^−1^ for mAb-SCoV2-S affinity interaction. The corresponding *K*_D_’s are 3.44 nM and 2.01 nM, respectively. The two-step consequent binding model was previously applied to other protein systems as granulocyte colony-stimulating factors receptors and ligands interaction [16] and to SARS-CoV-2 nucleocapsid protein and specific polyclonal antibody immune complex formation [15]. However, in these works, we used the TIRE method for obtaining interaction kinetics, and further analysis was performed using only mathematical equations, without taking into account viscoelastic properties. In the present study, we support our assumption of the two-step proteins monolayer formation not only by mathematical equations but also by experimentally obtained monolayer properties, such as viscosity and elasticity, that come from the application of advanced SE/QCM–D technique.

## 3. Materials and Methods

### 3.1. Materials and Reagents

11-Mercaptoundecanoic acid (11-MUA, 98%), 1-ethyl-3-(3-dimethylaminopropyl)carbodiimide (EDC, ≥98%), N-hydroxysuccinimide (NHS, 98%), ethanolamine (ETA) (≥99%) and phosphate-buffered saline (PBS) tablets were purchased from Sigma Aldrich. Methanol (99.9%) was purchased from Carl Roth GmbH & Co (Karlsruhe, Germany). Recombinant SCoV2-S glycoprotein expressed as secreted trimeric protein in mammalian (hamster) CHO cells was purchased from Baltymas (Vilnius, Lithuania). Gold-coated QCM–D quartz crystals with resonance frequency of 4.95 MHz and a diameter of 14 nm were purchased from Biolin Scientific (Gothenburg, Sweden). Specific monoclonal antibodies (mAb-SCoV2-S) were purchased from Sino Biological (Beijing, China).

### 3.2. Combined SE and QCM–D Measurement Setup

The measurement setup used consisted of a combination of SE and QCM–D. Ellipsometric measurements were performed with a spectroscopic ellipsometer with a rotating compensator M-2000X (J. A. Woolam, Lincoln, NE, USA) at a fixed angle of incidence of 65° in a spectral range of 200–1000 nm. The optical response was evaluated using the proprietary software Complete EASE (version 5.08gsc). QCM–D measurements i.e., frequency (Δ*F*) and dissipation (Δ*D*) were performed using a QSense Explorer (Biolin Scientific, Västra Frölunda, Sweden) operating at a fundamental frequency of 5 MHz and capable of simultaneously registering 7 harmonics up to ~65 MHz (13th harmonic). The measurements were performed in a special module for simultaneous SE/QCM-D signal monitoring. The QCM-D measurements were recorded with QSoft401 and analyzed with QSense Dfind. The flow of all fluids was regulated with an Ismatec IPC4 peristaltic pump (Cole-Palmer GmbH, Wertheim, Germany) connected via PTFE tubing to a fluid inlet through the measurement cell. Measurements were recorded under semi-static conditions with the fluid supply set at 1.35 mL/min. The temperature during the measurements was set to 20 °C.

### 3.3. Surface Preparation for Covalent SCoV2-S Immobilization

QCM–D sensors were plasma cleaned using Diener Femto Plasma Etcher (Ebhausen, Germany) in an oxygen environment for 1 min under 0.4 mbar pressure. The sensor was then immersed in a 1 mM 11-MUA solution in methanol for 18 h to form a self-assembled monolayer (SAM) of 11-MUA. The sensor was then rinsed with methanol and air-dried before being placed in the SE/QCM–D module. The module chamber was then filled with deionized water and a baseline was established after 30 min. To covalently immobilize the SCoV2-S molecules, the terminal carboxyl groups of 11-MUA SAM need to be activated. For this purpose, aqueous solutions of 0.1 M NHS and 0.4 M EDC were mixed in equal parts before being injected into the chamber for 15 min. Deionized water was then injected and a baseline was registered.

### 3.4. Covalent Immobilization of SCoV2-S and Affinity Interaction with mAb-SCoV2-S

After activation of the 11-MUA SAM carboxyl groups, a PBS solution was injected into the SE/QCM–D chamber for 10 min to establish a baseline. In the next step, 333 nM of SCoV2-S solution was injected for covalent SCoV2-S immobilization and incubated for 60 min. At the end of incubation, the immobilized SCoV2-S monolayer was washed with PBS solution. The surface was then further treated with 1 M ethanolamine hydrochloride solution (pH 8.5) for 10 min to block the remaining unbound activated carboxyl groups. Finally, the stabilized surface was washed once more with PBS solution. For SCoV2-S/mAb-SCoV2-S immune complex formation formed SCoV2-S monolayer was exposed to a 40 nM concentration mAb-SCoV2-S antibody solution for 60 min in order to form a monolayer of antibodies and ensure sufficient detectable signals. After that washing with PBS solution was performed.

## 4. Conclusions

A combined SE and QCM–D technique was used for the simultaneous investigation of SCoV2-S and mAb-SCoV2-S monolayers. The optical and mechanical properties reveal that the SCoV2-S monolayer is denser and more rigid than the mAb-SCoV2-S one. The kinetics of SCoV2-S and mAb-SCoV2-S monolayer formation can be well described with the two-step partial reversibility model, which assumes that the protein molecules are initially in an intermediate state that can either desorb or bind strongly. The analysis of SCoV2-S and mAb-SCoV2-S monolayer viscosity and elasticity time dependence during these monolayer’s formation process can be understood by assuming that the monolayers consist of two components, one of which is of relatively higher viscosity and corresponds to proteins in the intermediate state of two-step model, while the other component is significantly more rigid and can be associated with strongly bonded proteins. The results obtained suggest that the two-step SARS-CoV-2 spike protein and specific antibodies immune complex formation mechanism allows more precise evaluation of affinity constants and should be applied to study other corona viruses’ structural proteins and specific antibodies interactions as a model system.

## Data Availability

The data presented in this study are available on request from the first author.
